# A Micropatterning Strategy to Study Nuclear Mechanotransduction in Cells

**DOI:** 10.3390/mi10120810

**Published:** 2019-11-24

**Authors:** Markville Bautista, Anthony Fernandez, Fabien Pinaud

**Affiliations:** 1 Department of Chemistry, University of Southern California, Los Angeles, CA 90089, USA; mbbautis@usc.edu; 2 Department of Biological Sciences, University of Southern California, Los Angeles, CA 90089, USA; fernanam@usc.edu; 3 Department of Physics and Astronomy, University of Southern California, Los Angeles, CA 90089, USA

**Keywords:** surface silanization, cell micropatterning, emerin, lamin, Emery–Dreifuss muscular dystrophy (EDMD), nuclear shape index, mechanotransduction

## Abstract

Micropatterning techniques have been widely used in biology, particularly in studies involving cell adhesion and proliferation on different substrates. Cell micropatterning approaches are also increasingly employed as in vitro tools to investigate intracellular mechanotransduction processes. In this report, we examined how modulating cellular shapes on two-dimensional rectangular fibronectin micropatterns of different widths influences nuclear mechanotransduction mediated by emerin, a nuclear envelope protein implicated in Emery–Dreifuss muscular dystrophy (EDMD). Fibronectin microcontact printing was tested onto glass coverslips functionalized with three different silane reagents (hexamethyldisilazane (HMDS), (3-Aminopropyl)triethoxysilane (APTES) and (3-Glycidyloxypropyl)trimethoxysilane (GPTMS)) using a vapor-phase deposition method. We observed that HMDS provides the most reliable printing surface for cell micropatterning, notably because it forms a hydrophobic organosilane monolayer that favors the retainment of surface antifouling agents on the coverslips. We showed that, under specific mechanical cues, emerin-null human skin fibroblasts display a significantly more deformed nucleus than skin fibroblasts expressing wild type emerin, indicating that emerin plays a crucial role in nuclear adaptability to mechanical stresses. We further showed that proper nuclear responses to forces involve a significant relocation of emerin from the inner nuclear envelope towards the outer nuclear envelope and the endoplasmic reticulum membrane network. Cell micropatterning by fibronectin microcontact printing directly on HMDS-treated glass represents a simple approach to apply steady-state biophysical cues to cells and study their specific mechanobiology responses in vitro.

## 1. Introduction

Over the past two decades, micropatterning methods have become popular in biological studies. Initially used for the electronics industry, micropatterning now provides new tools for cell biology, and, combined with surface chemistry, it is particularly useful to study the interactions of cells with their microenvironment on different engineered surfaces [1,2]. Over the years, different micropatterning techniques of varying complexity have been developed, including microcontact printing, microfluidic patterning, UV-based deep etching and micro-stencils [3,4,5,6,7]. While there are advantages and drawbacks to each of these methods, they provide means to probe fundamental cellular functions, including cell adhesion and migration, cell polarity, cell shape dynamics, cytoskeletal rearrangement, and spatial coordination between cell and nuclear shape [1,8,9,10,11,12]. Microcontact printing has become one of the most commonly used micropatterning strategies because of its simplicity and robustness to generate micropatterns that guide cell adhesion and spreading. Moreover, the main advantage of this technique is that it only requires mild reagents, making micropatterning easily accessible to medical and biological laboratories. Microcontact printing is a soft lithographic technique based on the transfer of an “ink” from an elastomeric stamp to a surface [13]. Within a short contact time between the stamp and the surface, the “ink” is transferred with high precision and control. Microcontact printing has been utilized to transfer self-assembled monolayers of compounds such as alkylthiols on gold surfaces [14], while extra-cellular matrix proteins such as fibronectin or laminin have been used as “ink” to generate cell and tissue-compatible substrates [15,16,17].

Here, we tested different silanization strategies to optimize both microcontact printing of fibronectin on glass coverslips and cell micropatterning. We aimed at imposing specific physical strains at the cell nuclear envelope and at revealing some of the mechanotransducing functions of emerin in response to these mechanical cues. Emerin is a ubiquitous integral protein that is primarily located at the inner nuclear envelope (INE) and belongs to the LEM (LAP2, emerin, MAN1)-domain family of proteins [18,19]. It is encoded by the *EMD* gene and is composed of 254 amino acids with the LEM domain at the N-terminus, a large intrinsically disordered region, and a short C-terminal transmembrane domain [19]. Emerin participates in nuclear mechanotransduction and maintenance of the nuclear architecture by interacting with a variety of nucleoskeletal proteins such as lamins and nuclear actin, and with structural elements of the NE [19,20,21,22]. Nonsense mutations in the *EMD* gene and lack of emerin expression lead to Emery–Dreifuss muscular dystrophy (EDMD), an X-linked recessive disease [23] that causes degenerative skeletal muscle wasting, heart failure, and early death [24,25,26]. While the exact molecular mechanisms underlying the disease are still not fully established, one hypothesis to explain the muscle-specific phenotypes of EDMD [27] is the lost ability of the cell nucleus to adapt to mechanical cues due to the loss of emerin. For instance, isolated nuclei from emerin-null cells show defective nuclear adaptation to mechanical strains [28], whereas emerin-deficient mouse embryo fibroblasts display abnormal nuclear shape and impaired expression of mechanosensitive genes after mechanical challenges [21]. 

In this report, we demonstrate that two-dimensional cell micropatterning on rectangular fibronectin substrates of different widths can be efficiently employed to impose incremental physical strains at the cell nuclear envelope and to reveal some of the mechanotransducing responses of emerin. We show that changes in nuclear shape index in response to mechanical strains involve a relocation of emerin from the INE to the outer nuclear envelope (ONE) and the endoplasmic reticulum (ER).

## 2. Materials and Methods 

### 2.1. Surface Modification of Glass Coverslips

High precision microscope glass coverslips (Marienfeld, #1.5, Ø25 mm) were cleaned using a Piranha solution made of a 3:1 (*v*/*v*) mixture of 18 M sulfuric acid and 30% hydrogen peroxide for 15 min and rinsed thoroughly with deionized (DI) water. Following drying with nitrogen gas, the coverslips were heated to 95 °C in a sealed glass jar containing 100 μL of silane reagents for incremental reaction times of 3 to 90 min (silanization kinetics) or for a fixed reaction time of 90 min (cell micropatterning). The silane reagents used were: hexamethyldisilazane (HMDS, Sigma-Aldrich, St. Louis, MO, USA), (3-Aminopropyl)triethoxysilane (APTES, Sigma-Aldrich,) and (3-Glycidyloxypropyl)trimethoxysilane (GPTMS, Sigma-Aldrich). After vapor coating, the silane-coated coverslips were cleaned by sonication in water for 5 min and dried with N_2_ gas. The silane-treated coverslips were then stored in a separate and sealed glass container flushed with nitrogen. Static contact angle measurements were done on a ramé-hart 290-F1 Contact Angle Goniometer (ramé-hart instrument co., Succasunna, NJ, USA) with 5 μL water droplet volumes tested over 5–8 different positions per coverslip on at least 6 coverslips per reaction points. 

### 2.2. Fabrication of PDMS stamps

Polydimethylsiloxane (PDMS) stamps with rectangular micropatterns were produced from silicon masters fabricated using a chrome photomask (Minnesota Nano Center, Minneapolis, MN, USA). The micropatterns are 210 μm in length and have widths of 5 μm, 10 μm or 15 μm, respectively, with a constant periodic intervals of 30 μm. The etching depth on these silicon masters is 10 μm.

The silicon masters were treated with tridecafluoro-1,1,2,2-tetrahydrooctyl-1-trichlorosilane (Gelest Inc., Morrisville, PA, USA) for 90 min under vacuum to induce the formation of fluorosilane vapors and surface fluorosilanization of the masters. A 10:1 mixture of PDMS and curing agent (Sylgard 184 elastomer kit, Dow Corning, Midland, MI, USA) was combined in a plastic beaker and thoroughly mixed with a glass stirring rod for 10 min. As this step generates bubbles, the PDMS mixture is degassed under vacuum for 30 min (or by centrifugation at 3000 rpm for 10 min). The degassed PDMS mixture was then slowly poured onto the silicon masters, and another degassing step was done to avoid unwanted bubbles. After curing for 3 h at 65 °C and overnight at room temperature (RT), the PDMS stamps were removed slowly from the silicon master using a razor blade. Before being used in microcontact printing, the PDMS stamps were cleaned by 5 min sonication in water followed by 5 min sonication in ethanol. We routinely use the PDMS stamps for a period of 3–4 months without loss of stamping efficiency. 

### 2.3. Microcontact Printing

For microcontact printing, 100 μL of 100 mg/mL fibronectin in phosphate-buffered saline (PBS, 154 mM NaCl, 5.6 mM Na_2_HPO_4_, 1.1 mM KH_2_PO_4_, pH 7.5) were placed on the PDMS stamps and incubated at 25 °C for 30 min. The fibronectin solution was then aspirated off and the PDMS surface was rinsed twice with PBS. Upon drying, the inked PDMS surface was brought into contact with the silane-functionalized glass coverslips for 1–2 min, applying light pressure to guarantee good contact between the stamp and the glass. To block non-patterned areas, the coverslips were immersed in 1% (*m*/*v*) Pluronic F-127 (PF-127, Sigma-Aldrich) in Milli-Q water (EMD Millipore, Billerica, MA, USA) for 20 min, then rinsed three times with PBS prior to cell seeding.

To evaluate the quality of the stamping before and after PF-127 treatment, the fibronectin-stamped HMDS-, GPTMS- and APTES-coated coverslips were treated with 31 nM Cy3B-maleimide (GE Healthcare Life Sciences, Marlborough, MA, USA) in PBS for 1 h and rinsed three times with PBS, before imaging on a LSM 700 Confocal Laser Scanning Microscope (Zeiss, White Plains, NY, USA). To assess the antifouling efficacy of PF-127, 100 µL of 10 mg/mL (150 µM) of bovine serum albumin (BSA, Alfa Aesar-Thermo Fisher Scientific Chemicals, Ward Hill, MA, USA) was fluorescently labeled with 10 µL of 10 mg/mL (8 mM) of Alexa-Fluor 647 Succinimidyl Ester (Life Technologies, Carlsbad, CA, USA) for 1 h in PBS. The reaction was quenched with 150 µL of 20 mM Tris buffer (pH 7.8) for 1 h and the mixture was diluted to a final concentration of 2.5 mg/mL (37 µM) of A647-BSA with PBS. A647-BSA at 2.5 mg/mL was applied for 1 h on fibronectin-stamped coverslip previously labeled with Cy3B-maleimide and treated with PF-127. After three rinses with PBS, the coverslips were imaged by fluorescence confocal microscopy.

After stamping, the PDMS stamps were immersed in DI water and cleaned in an ultrasonic bath at 60 °C for 10 min, then immersed in 100% ethanol and sonicated for another 10 min at 60 °C before drying with nitrogen gas and storage. 

### 2.4. Cell Culture and Expression of Emerin Fusion

The cells used in this study are: human osteosarcoma U2OS cells, wild type human dermal fibroblasts (*Emd*^+/y^) and emerin-null human dermal fibroblasts (*Emd*^−/y^). All cells were maintained in Dulbecco’s modified Eagle’s medium (DMEM; Lonza, Walkersville, MD, USA) supplemented with 10% fetal bovine serum (FBS; Gibco-Life Technologies, Gaithersburg, MD, USA) and 1% penicillin/streptomycin (Lonza, Walkersville, MD, USA) in a humidified incubator at 37 °C, supplied with 5% CO_2_. For micropatterning, trypsinized cells resuspended in DMEM + 10% FBS were plated on fibronectin-stamped and PF-127-blocked coverslips, and were allowed to attach onto the micropatterns for 1 h. The cell culture media was then replaced in order to remove excess unattached cells. The cells were allowed to fully spread out for about 6 h at 37 °C before live imaging or chemical fixation.

Transduction of emerin in U2OS cells and in emerin-null human dermal fibroblasts was achieved with lentiviral particles containing a gene encoding a SNAP-tag N-terminal fusion to wild type emerin (SNAP-emerin). Cells grown at 70% confluence on 6 well-plates were infected with 150 µL of a viral titer at 0.09 µg/mL in complete medium containing 8 μg/mL polybrene for 48 h, after which the lentivirus was removed and replaced with fresh complete medium. After another 24-h incubation, cells were trypsinized and plated on micropatterns, as described above.

### 2.5. Immunofluorescence

For nuclear shape index (NSI) measurements, cells were fixed with 4% paraformaldehyde in PBS for 15 min, permeabilized with 0.1% Triton X-100 for 10 min, and blocked with 4% bovine serum albumin (BSA) + 0.1% Tween-20 for 1 h, all at RT. Wild type and emerin-null fibroblasts were then stained with Rhodamine Phalloidin (1:1000, Abcam Inc., Cambridge, MA, USA) for 1 h, and washed three times with PBS for 5 min each. Coverslips were mounted on a glass slide using DAPI-Fluoromount G (Electron Microscopy Sciences, Hatfield, PA, USA) and sealed with clear nailpolish. U2OS cells and emerin-null human dermal fibroblasts expressing SNAP-emerin were fixed, permeabilized, and blocked as mentioned. Before mounting in DAPI-Fluoromount G, cells were then additionally stained with 1:1000 of SNAP-Surface^®^ Alexa Fluor 647 benzylguanine substrate (BG-A647; New England Biolabs, Ipswich, MA, USA) in 4% BSA + 0.1% Tween-20 for 1 h at 37 °C and washed three times with PBS for 5 min each. Microscopy images were acquired by wide-field on an inverted Eclipse Ti-E microscope (Nikon Instruments Inc., Melville, NY, USA) or by confocal imaging on a Zeiss LSM 700 Confocal Laser Scanning Microscope.

For experiments involving emerin redistribution as a function of nuclear mechanical strains on micropatterns, wild type human dermal fibroblasts were fixed with 4% paraformaldehyde in PBS for 15 min, permeabilized with either 0.1% Triton X-100 (cell membrane and nuclear permeabilization, Sigma-Aldrich) or 0.1 % saponin (cell membrane permeabilization only, EMD Millipore) for 10 min, and blocked with 2% BSA + 1% normal goat serum (NGS) for 1 h, all at RT. Cells were stained with rabbit anti-emerin (1 μg/mL, Abcam Inc., Cambridge, MA, USA) and mouse anti-Lamin A/C (1:1000, Santa Cruz Biotechnology, Dallas, TX, USA) primary antibodies for 1 h at RT, then rinsed 3x with blocking buffer for 5 min. Staining with a goat anti rabbit-Alexa Fluor 488 (1:500, Life Technologies) and a goat anti mouse-Alexa Fluor 647 (1:1000, ThermoFisher, Life Technologies) secondary antibodies was then done for 1 h. Following three washing steps of 5 min in blocking buffer and three additional washing steps of 5 min in PBS, the coverslips were mounted and sealed as stated before. Images and z-scans through each cell were acquired on a Zeiss LSM 700 Confocal Laser Scanning Microscope. 

### 2.6. Microscopy Imaging

Confocal microscopy images were acquired on a Zeiss LSM 700 confocal laser scanning microscope equipped with a C-Apochromat 40×/1.2 W Korr objective, excitation lasers at 405 nm, 488 nm, 555 nm, and 639 nm, a multiband 405/488/555/639 beam splitter and appropriate emission filters for the detection of DAPI, Alexa Fluor 488, Cy3B, Rhodamine and Alexa Fluor 647. Images were acquired in 12-bit mode and the same settings were used across all samples. Confocal z-stacks were collected over the entire thickness of each cell in 0.34-μm slice intervals.

Wide field microscopy images were acquired on a Nikon Eclipse Ti-E microscope equipped with a 40× objective (Nikon), an X-Cite 120XL fluorescence illumination system, an iXon Ultra EMCCD camera (Andor) and appropriate filter sets for DAPI (Ex: 430DF24, Dich.:458DiO2,Em: 483DF32, Semrock) and A647 (Ex: 628DF40, Dich.:660DiO2,Em: 692DF40, Semrock) detections.

### 2.7. Image Analysis and Statistics

All image analyses were performed using FIJI software (version 1.52, National Institutes of Health, Bethesda, MD, USA). The nuclear shape index (NSI) [11] for each cell was determined by measuring the nuclear cross-sectional area and the nuclear perimeter of DAPI-stained nuclei, and by calculating the ratio:

(1) $$\mathrm{NSI}=\frac{4\times \pi \times \mathrm{area}}{{\mathrm{perimeter}}^{2}}.$$

The NSI is a measure of the roundness of the nucleus such that an NSI of 1 corresponds to a circular nuclear shape. Mean NSI values ± standard deviation of the mean are reported for n = 50–60 cell nuclei per condition.

Total ER and ONE emerin intensities were obtained from sum slices Z projection of each cell treated with saponin. After correction for background signal, the emerin intensity for each cell was normalized to the mean emerin intensity for non-patterned cells. ER to nuclear envelope emerin ratios were also measured from sum slices Z projection of cells treated with Triton X-100. For each cell, emerin intensities in the ER and the nuclear envelope were separately measured using region-of-interests drawn over the entire cell or over the cell nucleus only, using DAPI signal as a template. After correction for background signal, the ER to nuclear envelope intensity ratio for each cell was normalized to the mean ER to nuclear envelope intensity ratio for non-patterned cells. OriginPro (version 2019b, OriginLab, Northampton, MA, USA) was used to plot distribution, means and standard deviations of measured quantities. Statistical significances were assessed by two-tailed student’s *t*-tests. *p*-values < 0.05 were considered significant. 

## 3. Results 

### 3.1. Vapor-Phase Surface Silanization of Coverslip Surface

**Figure 1:**
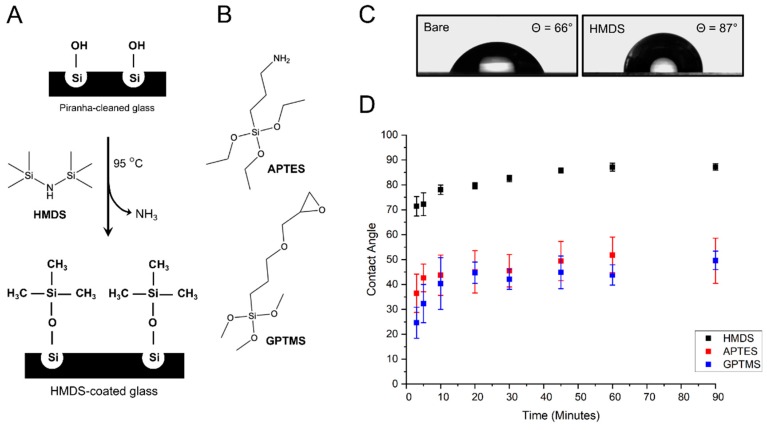
Glass coverslip silanization by vapor coating. (**A**) schematic representation of the vapor-phase silanization of glass coverslips with hexamethyldisilazane (HMDS); (**B**) structures of (3-Aminopropyl)triethoxysilane (APTES) and (3-Glycidyloxypropyl)trimethoxysilane (GPTMS); (**C**) optical image of water droplets taken by contact angle goniometry and showing significant difference in contact angle between bare glass coverslip and HMDS-coated coverslip; (**D**) kinetics of water contact angles for glass coverslips coated with different silane reagents.

We previously developed a method for surface functionalization of glass substrates via vapor phase deposition of monoreactive hexamethyldisilazane (HMDS) (Figure 1A) [29] and tested a similar silanization procedure with two commonly used silane reagents, 3-aminopropylethoxysilane (APTES) and (3-glycidyloxypropyl)trimethoxysilane (GPTMS) (Figure 1B). The efficiency of the silanization procedure can be assessed by water contact angle measurements. After cleaning in an ultrasonic bath and drying at 100 °C, bare glass coverslips display contact angles at about 66° (Figure 1C), indicative of a relatively good wettability. Upon activation of silanol group with Piranha solution, the contact angles dropped to <10° due to the presence of additional hydrophilic –OH groups on the glass surface [29]. Vapor silanization with HMDS renders the surface hydrophobic with the trimethysilyl groups forming single siloxane bonds on the silanol groups of the glass surface and subsequent release of NH_3_ gas. A kinetic study of HMDS deposition indicates that a complete glass surface coating is rapidly achieved within 45 to 90 min of reaction when contact angles saturate at 87° ± 1° (Figure 1D). These contact angle values are similar to those previously observed for monolayer depositions of HMDS on substrates [30]. They are also in very good agreement with the theoretical contact angle expected for a homogeneous monolayer of HMDS on glass and calculated based on the molecular cross-section of the trimethylsilyl group (Ф = 87°, Supporting Information). The HMDS-functionalized coverslips remained hydrophobic, without showing any change in contact angle for more than two weeks when stored under a dry N_2_ atmosphere.

For the vapor-phase deposition of GPTMS, water contact angles rapidly increase from 24° ± 6° after 3 min of reaction to 40° ± 10° at 10 min of reaction (Figure 1D), indicating an efficient attachment of GPTMS to the silanol groups on the glass surface, as previously observed by Tsukruk et al. [31]. As the reaction proceeds, contact angles continue to rise more slowly and gradually level off at 50° ± 4° after 90 min (Figure 1D). This second part of the silanization kinetic is similar to that reported for vapor and solution-phase self-assembled monolayer deposition of GPTMS on silicon wafers, where water contact angles saturate at around 52° once complete monolayer coverage is attained [31,32]. The reduction in the standard deviation of mean contact angles from 25% error at 10 min to 8% error at 90 min indicates that monolayer coverage becomes more homogeneous across the coverslip surface as the vapor-coating reaction proceeds. 

The kinetic of APTES deposition is similar to that of HMDS and GPTMS, although standard deviations of mean contact angle values are significantly higher (Figure 1D). This is likely due the fact that the terminal amino group of APTES can hydrogen bond with silanol groups, which provides more ways to interact with the glass surface and can lead to variation in the surface orientation of APTES compared to the two other organosilanes [33,34]. During APTES vapor-coating, contact angles rise rapidly to 45° ± 8° in the initial 20 min of reaction, after which the increase is significantly slower (Figure 1D). Ultimately, contact angles saturate in the range of 50° ± 9° after 90 min (Figure 1D), a value above the 41° contact angle expected for an APTES monolayer where the terminal amino groups homogeneously extend away from the glass surface [33,35]. In fact, the observed 50° ± 9° angles are consistent with an APTES coating where both hydrophilic amines and the more hydrophobic ethoxy groups are exposed due to inhomogeneous orientation of APTES molecules [33]. 

Thus, contrary to HMDS and GPTMS coatings where relatively homogeneous monolayer deposition is achieved, APTES deposition leads to more heterogenous and more hydrophobic glass surfaces than expected for an optimal monolayer coverage. Overall, HMDS monolayer deposition by vapor-coating makes for a significantly more hydrophobic and more homogeneous film on glass coverslips than similar deposition of GPTMS and APTES.

### 3.2. Cell Micropatterning

**Figure 2:**
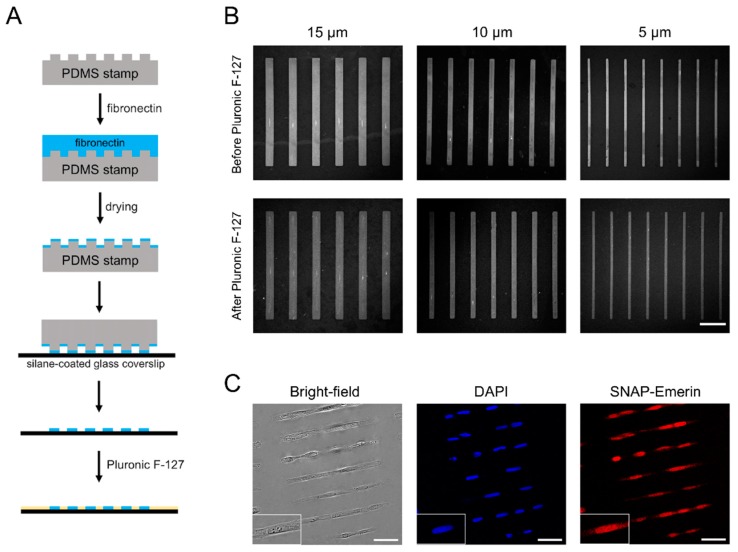
Fibronectin stamping and cell micropatterning on HMDS-coated coverslips. (**A**) schematic of microcontact printing of fibronectin on silane-modified glass substrates; (**B**) microscopy images of Cy3B-stained fibronectin strips on HMDS-coated coverslips before and after PF-127 treatment. Scale bar: 50 µm; (**C**) bright-field and wide-field fluorescence microscopy images of the nucleus (DAPI) and SNAP-emerin fusion for U2OS cells grown on 210 × 15 µm rectangular micropatterns. Scale bars: 50 µm.

We then tested if fibronectin is transferred efficiently by microcontact printing on coverslips functionalized with each type of organosilanes and if cells are effectively micropatterned on these substrates. Figure 2A summarizes the steps employed when preparing coverslips for cell micropatterning. A PDMS stamp containing rectangular micropatterns is incubated with fibronectin for 30 min. Upon drying and stamping onto the silane-functionalized glass coverslip, rectangular fibronectin islands are printed. Non-patterned areas are then blocked by physisorption of PF-127, a non-ionic triblock copolymer composed of a hydrophobic poly(propylene glycol) chain flanked by two poly(ethylene glycol) domains [29,36] that acts as an antifouling agent to prevent cell adhesion (Figure 2A). To determine the effectiveness and robustness of this stamping process, confocal images of the Cy3B-stained fibronectin patterns on HMDS-coated coverslips were taken before and after PF-127 treatment. 

For HMDS-coated coverslips, fibronectin was transferred efficiently and formed micropatterns having the same dimensions as those of the PDMS stamps (Figure 2B). Fibronectin remained firmly and specifically attached on the coverslips after incubation with PF-127 and following several washes in PBS to remove excess PF-127 (Figure 2B). To assess the microcontact printing quality across coverslips, the contrast value between “ON” stamped regions and “OFF” non-stamped regions was determined from fluorescence images of Cy3-labeled fibronectin. The contrast values for HMDS microstamping were 0.71 ± 0.04 before and 0.67 ± 0.07 after PF-127 treatment, indicative of a precise and reproducible fibronectin transfer (Table S1 in Supporting Information). U2OS cells seeded on the micropatterned coverslips, adhered specifically on the rectangular fibronectin islands within 30 min of incubation (Figure 2C) and remained fully spread out on the dimensions of the patterns after 6 h of incubation. No cell adhesion was observed in non-patterned and PF-127-blocked regions of the coverslips. During long-term culture, cells remained confined on the micropatterns for up to 72 h by alternating the use of serum-free cell media and daily 1–2 h feeding with 10% serum [29]. This robust transfer of fibronectin and the efficient physisorption of PF-127, which prevents cell adhesion and cell spreading outside micropatterns are due to the balanced surface wettability provided by the HMDS monolayer. Indeed, materials with water contact angles in the range of 80°–100° have adequate wettability [36] to promote both microcontact printing of the hydrophilic fibronectin and adsorption of the hydrophobic polypropylene glycol domain of PF-127 on surfaces. The homogeneous monolayer of hydrophobic trimethylsilyl groups in HMDS allow stable interactions with the polypropylene glycol of PF-127 and favor an outward orientation of its hydrophilic polyethylene glycol “arms” away from the glass surface to provide an efficient antifouling coating and block cell adhesion. Indeed, when we tested the antifouling efficacy of PF-127 on HMDS-coated coverslips using fluorescently labeled bovine serum albumin (A647-BSA), little to none non-specific binding of A647-BSA was observed outside fibronectin micropatterns (Figure S2 in Supporting Information), confirming the strong anchoring of PF-127 on HMDS. Note that HMDS alone, while making the surface hydrophobic, does not block cell adhesion without the use of PF-127.

**Figure 3:**
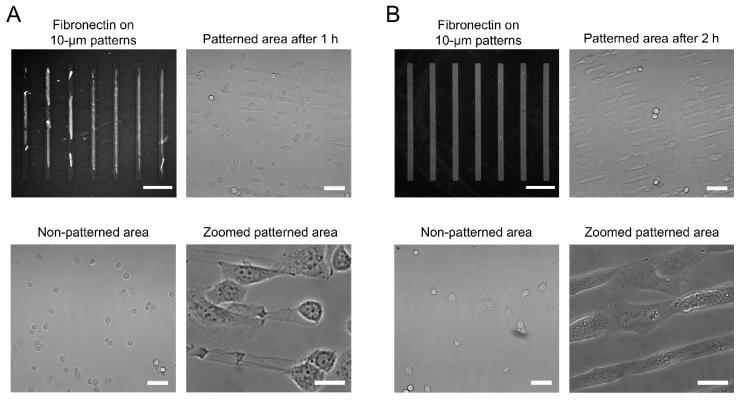
Fibronectin stamping and cell micropatterning on APTES- and GPTMS-coated coverslips. (**A**) microscopy images of Cy3B-stained fibronectin strips on APTES-coated coverslips after PF-127 treatment and of cell distribution in micropatterned and non-micropatterned areas; (**B**) microscopy images of Cy3B-stained fibronectin strips on GPTMS-coated coverslips after PF-127 treatment and of cell distribution in micropatterned and non-micropatterned areas. For both A and B, scale bars: 50 µm (fibronectin patterns), 100 µm (patterned and non-patterned areas), 20 µm (zoomed areas).

For the APTES- and GPTMS-coated coverslips, fibronectin can also be inked to the glass surfaces, where it forms the expected 210 × 10 µm rectangular micropatterns (Figure S1). However, the fibronectin transfer is not as homogeneous as for the HMDS-coated surfaces, with contrast values of 0.57 ± 0.10 for APTES and 0.79 ± 0.11 for GPTMS, both of which have significantly larger standard deviations than for HMDS (Figure S1 and Table S1 in Supporting Information). After PF-127 treatment, significant desorption of the stamped fibronectin is observed for the APTES-coated coverslips and the rectangular micropatterns become very imprecise, with a contrast value dropping to 0.28 ± 0.11 (Figure 3A and Table S1 in Supporting Information). Because of this loss in surface fibronectin, cells seeded on APTES-coated coverslips are not reliably confined to the expected rectangular patterns (Figure 3A). In addition, many cells attach to non-stamped areas of the coverslips, indicative of an inefficient surface antifouling by PF-127 (Figure 3A). Consistent with the inability of PF-127 to act as an efficient antifouling agent, A647-BSA displays significantly more non-specific binding outside micropatterned areas on APTES-coated coverslips than on HMDS-coated coverslips (Figure S2 in Supporting Information).

For GPTMS-coated coverslips, we also observed a partial loss of fibronectin upon incubation with PF-127, but the rectangular micropatterns remained well defined, with a contrast value of 0.72 ± 0.06 (Figure 3B and Table S1 in Supporting Information). While cells seeded on GPTMS-coated coverslips adhere to the rectangular fibronectin islands and adapt their overall shape to the dimensions of the patterns, many cells also bind to non-stamped area (Figure 3B). Despite incubation with PF-127, micropatterned cells rapidly escape the confinement of the micropatterns and start growing outside the fibronectin ink 2 h after seeding (Figure 3B). Indeed, as for APTES-coated coverslips, A647-BSA exhibits relatively high non-specific binding outside the fibronectin micropatterns, indicating that PF-127 is not well attached to the GPTMS surface or insufficiently well oriented to exhibit efficient antifouling properties (Figure S2 in Supporting Information). In contrast, we see no attachment of cells outside micropatterns and no escape from fibronectin islands even 72 h after cell seeding on HMDS-treated coverslips. Compared to HMDS-coated glass, the higher surface wettability of GPTMS- and APTES-coated coverslips, outside the optimal 80°–100° contact angle range [36], likely explains the inefficient physisorption of PF-127 and the issues in controlling long-term confinement of cells within the fibronectin micropatterns. 

These data indicate that HMDS-treated coverslips provide a much more robust and reproducible surface for cell patterning compared to GPTMS or APTES coatings. Indeed, the balanced wettability of the HMDS monolayer not only allows homogeneous microcontact printing of extra-cellular matrix proteins, but also provides a good physisorption of the PF-127 antifouling agent that prevents cell adhesion outside patterns and limits cell escape from microstamped areas over long periods of time. 

### 3.3. Micropatterning to Study Emerin Mechanotransduction

**Figure 4:**
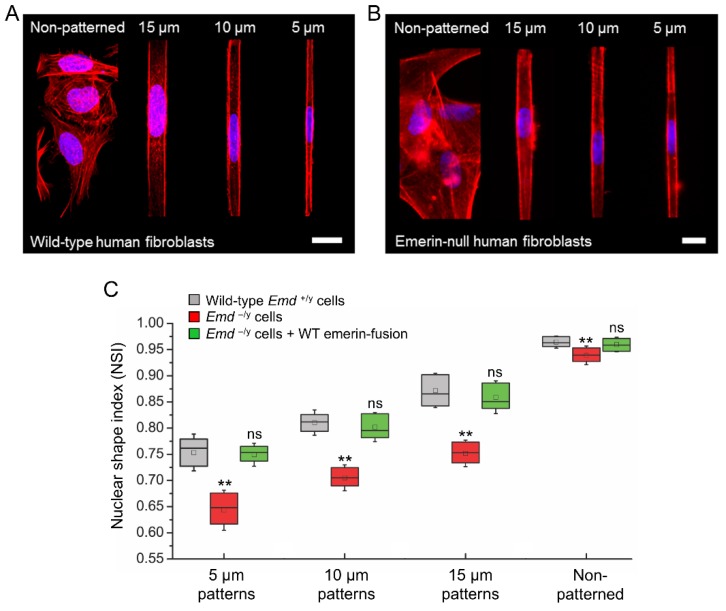
Applying mechanical strains to the nucleus by micropatterning cells on increasingly narrow rectangular fibronectin strips. (**A**) confocal images of wild type fibroblasts immunostained for F-actin (red) and the nucleus (blue) after plating on non-patterned or patterned rectangular fibronectin substrates having 15 µm, 10 µm and 5 µm widths. Scale bar: 20 µm; (**B**) wide-field images of emerin-null fibroblasts immunostained for F-actin (red) and the nucleus (blue) after plating on non-patterned or patterned rectangular fibronectin substrates having 15 µm, 10 µm and 5 µm widths. Scale bar: 20 µm, (**C**) changes in NSI as a function of micropattern width for wild type human skin fibroblasts (*Emd*^+/y^), emerin-null skin fibroblasts (*Emd*^−/y^) and emerin-null skin fibroblasts rescued with wild type (WT) SNAP-emerin fusion (*Emd*^−/y^ + WT emerin-fusion). The box length represents the NSI interquartile range, the central square represents the NSI mean, the central bar represents the NSI median, and the error bars represent the standard deviation of the mean. N = 50–60 cell nuclei per condition. *T*-test comparison to *Emd*^+/y^ cells: ** *p* < 0.01, ns: non-significant.

We then used cell micropatterning to evaluate some of the mechanotransducing functions of emerin, an INE protein linked to EDMD. While emerin is expressed in all somatic cells, nonsense mutations in the *Emd* gene and absence of emerin expression primarily affect cells exposed to extensive mechanical stress, such as skeletal and cardiac muscle cells [37]. We thus studied the influence of increasing mechanical strains on the nuclear shape of wild type (*Emd*^+/y^) and emerin null (*Emd*^−/y^) human skin fibroblasts by patterning them on increasingly narrow rectangular fibronectin islands inked on HMDS-coated coverslips (Figure 4A,B). These fibronectin micropatterns have widths of 15, 10, or 5 µm and constrain adhesion to regions smaller than the size of the fibroblasts, effectively resulting in cell-shape modification and actin-driven modulation of the nuclear shape index (NSI) [11,29]. The NSI is a measure of the roundness of the nucleus such that an NSI of 1 corresponds to a circular nuclear shape [11]. As shown in Figure 4C, *Emd*^+/y^ fibroblasts have a nearly circular nucleus with NSI of 0.96 ± 0.01 when grown randomly on fibronectin. As nuclear mechanical strains increase, the nucleus adopts an oval shape and the NSI of *Emd*^+/y^ fibroblasts gradually decreases to 0.88 ± 0.03, 0.81 ± 0.02 and 0.76 ± 0.03 for 15, 10, and 5 µm-wide patterns, respectively (Figure 4A,C). In comparison, *Emd*^−/y^ fibroblasts from EDMD patients grown randomly on fibronectin display a slightly more deformed nucleus with an NSI at 0.94 ± 0.01 (*p* < 0.01, Figure 4B,C), consistent with previous results in emerin-deficient mouse fibroblasts [21] and human muscle cells [38]. The increase nuclear deformation and modified nuclear envelope stiffness of *Emd*^−/y^ fibroblasts are more evident from the NSI values obtained in micropatterns, all of which are significantly lower than those of normal *Emd*^+/y^ fibroblasts (*p* < 0.01, Figure 4C). 

These aberrant nuclear mechanoresponses of emerin-null fibroblasts are directly linked to the absence of emerin. Indeed, rescuing emerin expression in *Emd*^−/y^ cells by lentiviral transduction of a SNAP-tag N-terminal fusion to wild type emerin (SNAP-emerin) results in a full recovery of normal nuclear responses to forces, with no significant difference in NSI compared to wild type *Emd*^+/y^ fibroblasts (ns, Figure 4C). These results confirm that human cells lacking emerin expression have abnormal nuclear envelope mechanics and that emerin critically participates in maintenance of the nuclear architecture. 

**Figure 5:**
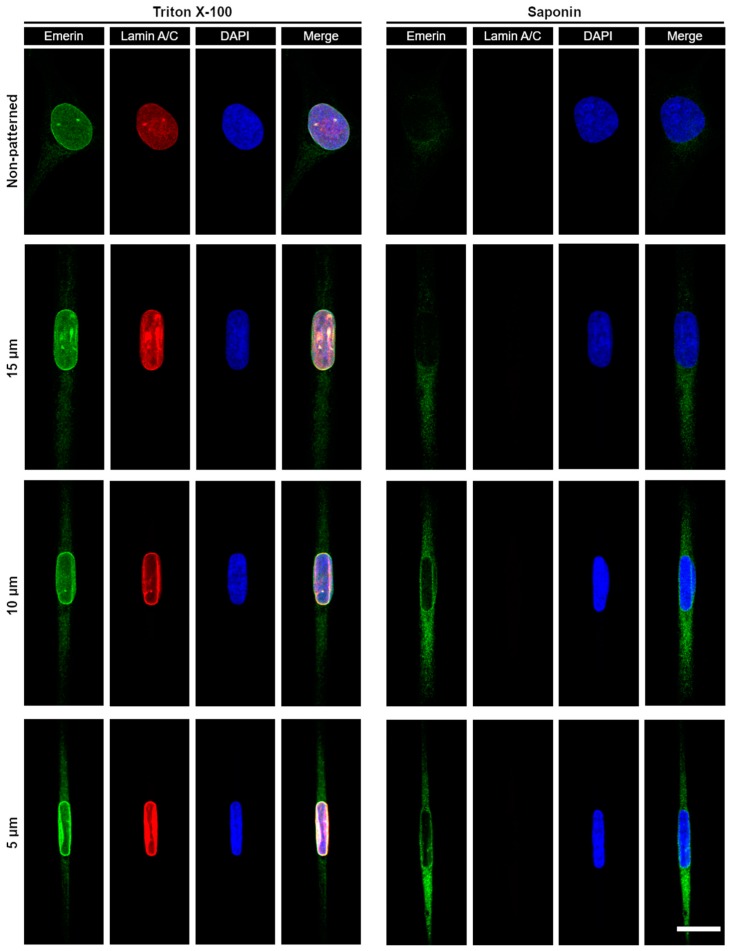
Fluorescence confocal imaging of emerin distribution in wild type *Emd*^+/y^ human skin fibroblasts as a function of nuclear strains. The distribution of the entire cellular pool of emerin (ER, ONE, and INE) is imaged in cells treated with Triton X-100 as a detergent to permeabilize both the plasma and the nuclear membrane (left panels). The pool of emerin only associated with the ER and the ONE is imaged in cells treated with saponin to permeabilize only the plasma membrane (right panels). Cells grown randomly on fibronectin (non-patterned) or on increasingly narrow rectangular micropatterns (15–5 µm) are immunostained for emerin (green), lamin A/C (red) and the nucleus (blue). Scale bar for all images: 20 µm.

**Figure 6:**
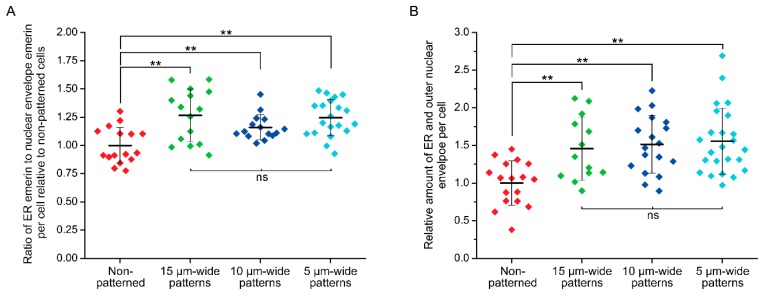
Quantification of emerin redistribution in response to nuclear mechanical strains. (**A**) ratio of ER emerin to nuclear envelope (ONE and INE) emerin for non-patterned *Emd*^+/y^ skin fibroblasts (Triton X-100, *n* = 16 cells) or fibroblasts grown on 15 µm-wide (Triton X-100, *n* = 15 cells), 10 µm-wide (Triton X-100, *n* = 15 cells), and 5 µm-wide (Triton X-100, *n* = 20 cells) fibronectin rectangular micropatterns. The fluorescence intensity quantification is done on sum slices images of full cell confocal z-scans obtained after Triton X-100 permeabilization and emerin immunostaining. For each cell, the intensity ratio is normalized to the mean ER/nuclear envelope ratio of non-patterned cells; (**B**) relative amount of ER and outer nuclear envelope emerin for non-patterned *Emd*^+/y^ skin fibroblasts (saponin, *n* = 17 cells) or fibroblasts grown on 15 µm-wide (saponin, *n* = 14 cells), 10 µm-wide (saponin, *n* = 18 cells), and 5 µm-wide (saponin, *n* = 24 cells) fibronectin rectangular micropatterns. The fluorescence intensity quantification is done on sum slices images of full cell confocal z-scans obtained after saponin permeabilization and emerin immunostaining. For each cell, the quantified emerin intensity is normalized to the mean emerin intensity for non-patterned cells. For both (A) and (B), the thick bars and the error bars represent the mean and the standard deviation of each distribution, respectively. T-tests: ** *p* < 0.01, ns: non-significant.

While emerin primarily localizes at the INE, it was recently shown that external forces cyclically applied to the nucleus of epidermal keratinocytes trigger a partial redistribution of emerin to the ONE [39]. When cells are patterned on narrow fibronectin strips, we also previously observed [29] that transiently expressed emerin fusions have increased association with the perinuclear ER membrane, which is continuous with the ONE. We therefore determined if deformation of the nuclear envelope and maintenance of nuclear shape against steady-state mechanical strains imposed by our micropatterns would also implicate a relocation of endogenous emerin from the INE toward the ONE and the ER membrane. The distribution of emerin was studied in wild type *Emd*^+/y^ skin fibroblasts micropatterned on increasingly narrow rectangular fibronectin islands using cell permeabilization with Triton-X100 to image the entire cellular pool of ER, ONE, and INE emerin or with saponin to image only the ER and ONE pools (Figure 5). While fibroblasts grown randomly on fibronectin display a clear enrichment of emerin at the INE with seldom ER and ONE emerin, mechanical strains and ensuing nuclear deformations are accompanied by a non-negligible relocation of emerin to the ER and the ONE (Figure 5). A quantitative analysis of cells treated with Triton X-100, reveals that the ratio of ER to total nuclear envelope emerin increases by about 25% as the nucleus adapts to strains (*p* < 0.01, Figure 6A). This increase remains constant across different nuclear deformations with no significant changes between 15, 10, and 5 µm patterns (Figure 6A). These results indicate that adjusting nuclear shapes against mechanical strains involves an initial relocation of emerin to the ER membrane independent of the overall emerin expression level. A similar analysis of cells permeabilized with saponin confirms that the amount of endogenous ER and ONE emerin increases significantly when the nucleus is subjected to mechanical strains in micropatterns (Figure 6B). Specifically, the amount of emerin associated with the ER and the ONE grows by 50% in micropatterned fibroblasts, with normalized emerin quantities shifting from 1.00 ± 0.29 (*n* = 17 cells) for non-patterned fibroblasts to 1.46 ± 0.42 (*n* = 13 cells, *p* < 0.01), 1.52 ± 0.38 (*n* = 18 cells, *p* < 0.01) and 1.56 ± 0.44 (*n* = 24 cells, *p* < 0.01) for fibroblasts on 15, 10, and 5 µm-wide patterns, respectively (Figure 6B). Together, these data demonstrate that a key mechanotransducing function of emerin is to partially relocate from the INE to the ONE and the ER membrane in order to guarante adequate nuclear deformation when a cell nucleus is exposed to mechanical challenges.

## 4. Conclusions

Through a simple micropatterning strategy, we investigated the function of emerin during nuclear mechanoresponses in human skin fibroblasts. We demonstrated that HMDS monolayer deposition by vapor coating on glass coverslips provides a balanced surface wettability, with sufficient hydrophilicity for microstamping of fibronectin and adequate hydrophobicity to maintain PF-127 on non-stamped areas and provide good anti-fouling efficiency. While cells might be shaped in many different geometries on two-dimensional micropatterns, robust and stable cell confinement in rectangular fibronectin islands having widths smaller than the dimension of a typical cell provides a simple means to impose and modulate steady-state mechanical strains on the nucleus, as demonstrated by gradual changes in NSI. Importantly, the parallelization provided by micropatterning allows these forces to be applied homogeneously and simultaneously over hundreds of cells. Using different size micropatterns, we showed that cellular expression of emerin is critical for the maintenance of nuclear shape against increasing mechanical strains. We also demonstrated that nuclear deformation against forces is associated with an initial redistribution of emerin from the INE towards the ONE and the ER membrane. These results confirm that emerin is a key element of nuclear mechanotransduction processes at the nuclear envelope. Additional micropatterning studies with cells expressing EDMD-inducing emerin mutants will likely provide new insights into the pathogenesis of EDMD and the mechanobiological functions of emerin and the nucleus. Overall, cell micropatterning by direct fibronectin microcontact printing on vapor-coated HMDS coverslips provide a straightforward approach to impose steady-state forces to cells and study their molecular mechanobiology in a quantitative manner in vitro.

## Supplementary Materials

The following are available online at https://www.mdpi.com/2072-666X/10/12/810/s1, Figure S1: Effect of PF-127 treatment on fibronectin attachment to APTES- and GPTMS-coated coverslips, Figure S2: Antifouling efficacy of PF-127 treatment on HMDS-, APTES- and GPTMS-coated coverslips after fibronectin attachment, Table S1: Quality assessment of fibronectin micropatterns.
