# Supplementary material for: A Micropatterning Strategy to Study Nuclear Mechanotransduction in Cells
Source: Micromachines (Basel). 2019 Nov 24;10(12):810. doi: 10.3390/mi10120810 (PMC6952994; doi:10.3390/mi10120810)
Supplement: Supplementary file 1 [file micromachines-10-00810-s001.pdf]

Supplementary Materials: A Micropatterning Strategy to Study Nuclear Mechanotransduction in Cells

Markville Bautista, Anthony Fernandez and Fabien Pinaud

1. Calculation of Theoretical Contact Angle for a Monolayer Formation with HMDS

The Cassie equation [1] describes the expected contact angle of a solute on a bifunctional surface (in our case the piranha-treated coverslip glass surface and its HMDS layer) as follows:

$$\cos \Phi = f_1 \cos \theta_1 + f_2 \cos \theta_2, \quad (1)$$

where Φ is the equilibrium contact angle of water on the bifunctional surface (glass coverslip + HMDS), f_1 is the molecular fraction coverage of HMDS on the bifunctional surface, θ_1 is the expected contact angle of water on a pure HMDS surface (without coverslip support), f_2 is the molecular fraction of the bifunctional surface not covered by HMDS, and θ_2 is the expected contact angle of water on the pure glass coverslip surface (without HMDS).

f_1 and f_2 depend:

(i) On the circular cross-sectional area taken up by the trimethylsilyl group ($Si\text{-}(CH_3)_3$) of HMDS, which has been estimated by Herzberg et al. [2] at 27.7 Å^2

and

(ii) On the number per surface area of hydroxyl silanol groups (Si-OH) that are available to react with HMDS on the coverslips. For a fully hydroxylated silica surface, this number has been estimated at 5 per 100 Å^2 by different groups [3–5], and it is assumed to be unchanged for glass.

Thus, for a perfectly modified glass coverslip substrate where coverage and reaction of HMDS with silanol groups is optimal (monolayer), the surface molecular fraction coverage of trimethylsilyl should be f_1 = (100/27.7)/5 = 0.722. Consequently, we have a glass surface molecular fraction non-covered by HMDS of f_2 = 1 − 0.722 = 0.278.

The contact angle value of water on the piranha-treated glass coverslip surface (without HMDS) was measured and reported in Fernandez et al. [6] as θ_2 = 3.5°. There is, however, no known $\theta 1$ value for a pure HMDS surface (without substrate support) because HMDS cannot polymerize on its own into a solid surface. However, the contact angle value of water on PDMS can be used as an appropriate estimate of θ_1 for HMDS because this polymer is extremely rich in methyl groups similar to those found in HMDS. Here, we used a contact angle of water on PDMS of 108° as determined by molecular simulation [7]. This value is in good agreement with reported experimental estimates between 98° and 112° (see references in Ismail et al. [7]). Thus, with values: f_1 = 0.72, θ_1 = 108°, f_2 = 0.28 and θ_2 = 3.5° we can evaluate the expected equilibrium contact angle of water (Φ) for a perfect and monolayer coverage of HMDS on a glass coverslip, from Cassie's equation. We obtain cos Φ = 0.054 and Φ = 87°. This value is in excellent agreement with our measured value of 87 ± 1° after 90 min of reaction, thus indicating that vapor coating provide a homogenous monolayer deposition of HMDS on glass coverslips.

We note that multilayer polymerization of HMDS at the coverslip surface (although it is improbable considering the nature of the chemical reaction) would result in water contact angles that would increase toward 108° overtime. Similarly, sub-monolayer coverage would result in water contact angles that would decrease from the theoretical 87° value towards the 3.5° angle value measured for piranha-treated glass.

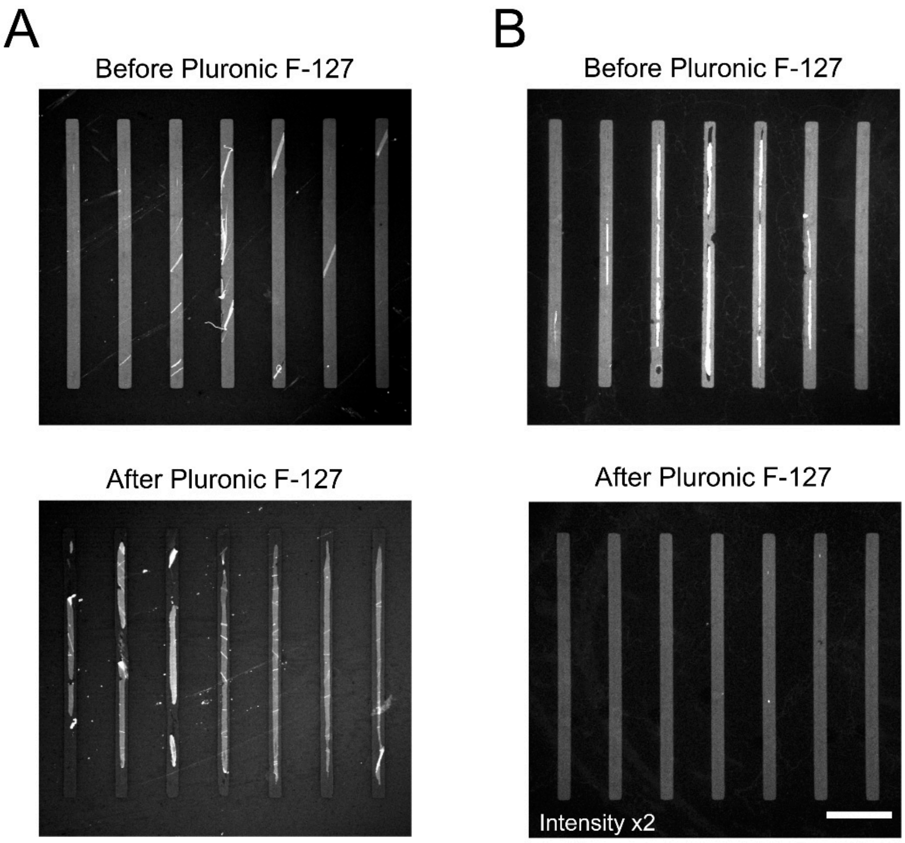

Figure S1. Effect of PF-127 treatment on fibronectin attachment to APTES- and GPTMS-coated coverslips. (**A**) fluorescence confocal images of fibronectin microstamped from 210 × 10 µm rectangular PDMS stamps on APTES-coated coverslips before and after treatment with PF-127; (**B**) fluorescence confocal images of fibronectin microstamped from 210 × 10 µm rectangular PDMS stamps on GPTMS-coated coverslips before and after treatment with PF-127. Note that the fluorescence intensity in the microscopy image after pluronic treatment for GPTMS was multiplied by two to facilitate a visualization of the micropatterns. Scale bar for all images: 50 µm.

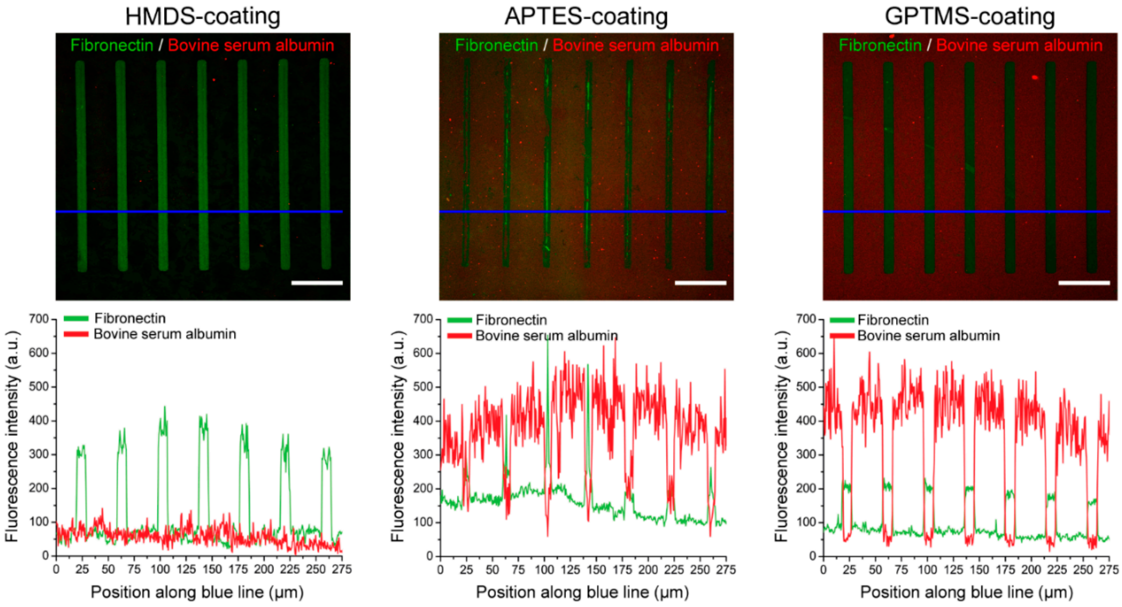

Figure S2. Antifouling efficacy of PF-127 treatment on HMDS-, APTES- and GPTMS-coated coverslips after fibronectin attachment. **Top row:** Dual-color fluorescence confocal images of fibronectin microstamped from 210 × 10 µm rectangular PDMS stamps (green) and of A647-BSA non-specifically

binding outside micropatterns (red) on HMDS-, APTES- and GPTMS-coated coverslips after treatment with PF-127. All scale bars: 50 μm. **Bottom row:** Fluorescence intensity profiles for fluorescently labeled fibronectin (green) and A647-BSA (red) along the blue line in each of the corresponding confocal images from the top row.

2. Measurements of Contrast Values to Compare the Quality of Fibronectin Microcontact Printing across Coverslips Coated with Different Organosilanes

To quantify the quality of the fibronectin micropatterns, fluorescence intensities of Cy3B-fibronectin along microcontact printed strips (n = 15–25 rectangular 10 × 210 μm strips) on multiple coverslips were measured for the three organosilane coatings, before and after PF-127 treatments. Specifically, the mean contrast fluorescence values ± standard deviation were calculated as previously described [8] as:

$$C = \frac{I_{\text{on}} - I_{\text{off}}}{I_{\text{on}}}, \quad (2)$$

where C is the mean contrast value, *Ion* is the mean fluorescence intensity within the rectangular fibronectin strips where fibronectin transfer occurs, and I_{off} is the mean fluorescence intensity in-between strips, where no transfer should occur. These results are reported in Supporting Table S1.

Table S1. Quality assessment of fibronectin micropatterns.

Coating	Contrast Value ± Standard Deviation	
	Before Pluronic F127	After Pluronic F127
HMDS	0.71 ± 0.04	0.67 ± 0.07
APTES	0.57 ± 0.10	0.28 ± 0.11
GPTMS	0.79 ± 0.11	0.72 ± 0.06

References

1. Cassie, A.B.D. Contact angles. *Discuss. Faraday Soc.* **1948**, *3*, 11–16.
2. Herzberg, W.J.; Marian, J.E.; Vermeulen, T. Receding contact angle. *J. Colloid Interface Sci.* **1970**, *33*, 164–171.
3. Davydov, V.Y.; Kiselev, A.V.; Zhuravlev, L.T. Study of the surface and bulk hydroxyl groups of silica by infra-red spectra and d2o-exchange. *Trans. Faraday Soc.* **1964**, *60*, 2254–2264.
4. Zhuravlev, L.T. The surface chemistry of amorphous silica. Zhuravlev model. *Colloids Surf. A* **2000**, *173*, 1–38.
5. Zhuravlev, L.T. Concentration of hydroxyl groups on the surface of amorphous silicas. *Langmuir* **1987**, *3*, 316–318.
6. Fernandez, A.; Bautista, M.; Stanciauskas, R.; Chung, T.; Pinaud, F. Cell-shaping micropatterns for quantitative super-resolution microscopy imaging of membrane mechanosensing proteins. *ACS Appl. Mater. Interfaces* **2017**, *9*, 27575–27586.
7. Ismail, A.E.; Grest, G.S.; Heine, D.R.; Stevens, M.J.; Tsige, M. Interfacial structure and dynamics of siloxane systems: Pdms-vapor and pdms-water. *Macromolecules* **2009**, *42*, 3186–3194.
8. Lindner, M.; Tresztenyak, A.; Fülöp, G.; Jahr, W.; Prinz, A.; Prinz, I.; Danzl, J.G.; Schütz, G.J.; Sevcsik, E. A fast and simple contact printing approach to generate 2d protein nanopatterns. *Front. Chem.* **2019**, *6*, 655–655.
